# Association Between Length of Stay and Incidence of Hospital-Acquired Anaemia in Critically Ill Patients: A Retrospective Cohort Study

**DOI:** 10.1155/ccrp/8884182

**Published:** 2025-05-27

**Authors:** Bushra Al Amer, Ghaleb Alharbi, Abdulaziz Alrashdi, Hameed Alrashedi, Majd Alsaeed, Razan Almahubi, Yara Almarshad

**Affiliations:** ^1^College of Pharmacy, Qassim University, Buraydah, Qassim, Saudi Arabia; ^2^Department of Clinical Pharmacy, College of Pharmacy, Shaqra University, Shaqra 11961, Saudi Arabia; ^3^Department of Pharmacology and Toxicology, College of Pharmacy, Qassim University, Qassim, Saudi Arabia; ^4^King Saud Hospital, Ministry of Health, Riyadh, Qassim, Saudi Arabia; ^5^Innova Health House, Buraydah, Qassim, Saudi Arabia; ^6^Procter & Gamble, Riyadh, Saudi Arabia

**Keywords:** anaemia, bleeding, critical care, haemoglobin, hospital-acquired anaemia, intensive care unit, iron deficiency

## Abstract

Hospital-acquired anaemia (HAA) is characterised by initially normal haemoglobin levels upon admission that are lowered during the hospital stay. The decreased haemoglobin levels related to the days of intensive care unit (ICU) hospitalisation may explain the effect of other interventions on haemoglobin levels. This study aimed to investigate the association between decreased haemoglobin levels and days of hospitalisation in critically ill patients in the Qassim region by analysing haemoglobin levels within the first 7, 14, and 21 days after ICU admission. A total of 180 patients were admitted during the study period. Patients with gastrointestinal bleeding, transfusion-dependent anaemia, a history of anaemia or bleeding, those with chronic kidney disease or on dialysis, and those who had hematologic or other malignancies were excluded (*n* = 97). Finally, those who were at least 18 years old, was within the normal range of haemoglobin upon admission to the ICU and had been hospitalized for at least 21 days in the ICU were included (*n* = 83). The initial average haemoglobin concentration was higher in men (15.24 g/dL) than in women (13.45 g/dL). Both experienced a significant and relatively parallel decline in haemoglobin levels (8.95 g/dL) and (8.66 g/dL), respectively, throughout the 21 day hospitalization period. The *p* value (< 0.001) suggests that the fixed effects are statistically significant, indicating that time (days) has a significant effect on haemoglobin levels. This study found a consistent decrease in haemoglobin levels over the ICU hospitalisation period, suggesting a progressive condition or treatment effect leading to reduced haemoglobin levels. However, further studies are required to analyse the causes of HAA in ICU.

## 1. Introduction

Hospital-acquired anaemia (HAA) is characterised by initially normal haemoglobin levels upon admission that later reduce during the hospital stay [1]. Anemia is described as a reduction in the proportion of the red blood cells [2].

Normal Haemoglobin (Hgb)-specific laboratory cut-offs will differ slightly, but in general, the normal ranges are as follows: 13.5 to 18.0 g/dL in men, 12.0 to 15.0 g/dL in women, 11.0 to 16.0 g/dL in children [2]. When the hemoglobin level is low, the patient has *anemia* The development of anaemia in critical illness can be attributed to several factors, such as surgical procedures leading to the loss of red blood cells, bleeding, trauma, or gastrointestinal bleeding; reduced production caused by inflammatory cytokines, medications, or renal dysfunction; nutritional deficiencies, such as iron, folic acid, and vitamin B12; and heightened destruction of red blood cells or their precursors in the bone marrow due to toxins and medications. In the intensive care unit (ICU), anaemia is linked to negative results, such as failure of liberation from mechanical ventilation, type 2 myocardial infarction, and a higher likelihood of death. Anaemia can also result in incorrect measurement of blood sugar levels, which can lead to a higher risk of low blood sugar when determining the insulin dosage. Long-lasting anaemia following discharge from the ICU could have lasting effects [3]. The haemoglobin level throughout ICU admission varies according to the patient's disease, risk factors, and procedures performed. We hypothesized whether there was an association between haemoglobin levels and the length of stay in the ICU in critically ill patients in the Qassim region of Saudi Arabia. Our study aimed to analyse variations in haemoglobin concentrations within the first 7, 14, and 21 days of ICU admission.

## 2. Materials and Methods

### 2.1. Study Design, Population, and Data Sources

A retrospective observational cohort analysis was conducted on all patients admitted to the ICU between October 2023 and December 2023. The ICU setting is a 52-bed mixed surgical-medical unit located in a referral hospital, the King Fahad Specialist Hospital, in the Qassim region. A total of 180 patients were admitted during the study period. We excluded patients with gastrointestinal bleeding, transfusion-dependent anaemia, a history of anaemia, haemolysis, haemorrhagic stroke, or retroperitoneal bleeding. Additionally, those with chronic kidney disease, those on dialysis, those with haematologic malignancies, or those who were treated with medications such as iron, erythropoiesis-stimulating agents, or chemotherapy were also excluded. Individuals who had central or peripherally inserted central line placement; received a blood transfusion during hospitalisation; were hospitalised for less than 21 days, or were assigned to surgery, cardiac care units, or step-down units were excluded. Furthermore, patients with haemoglobin levels in the anaemic range (< 13 g/dL for men and < 12 g/dL for women, according to WHO criteria) upon ICU admission were also excluded from the study. We included patients who were at least 18 years old, had normal haemoglobin concentrations upon admission to the ICU, and were hospitalised for at least 21 days in the ICU, totalling 83 patients. By using detailed exclusion measures we succeeded to demonstrate how time-related changes largely contribute together with patient-specific factors to the overall variability in decreasing of hemoglobin levels during ICU hospitalization.

Worth mentioning that some minor procedures have an impact on the deficiency of participants hemoglobin caused by weakness of the documentation it was difficult to list them.

Demographic and haemoglobin data were retrieved from the electronic medical record system. HAA was defined as having a haemoglobin level lower than the WHO's sex-specific cut points normal range (13.8 to 17.2 g/dL for men and 12.1 to 15.1 g/dL for women) after the first 24 h of admission [3].

### 2.2. Study Dates

#### 2.2.1. Actual Study Day

The actual study day is the day where participants were enrolled into the study regardless of the actual admission day to the hospital. Each participant's actual study day was calculated as follows: the reference date (Day 1) was the day of the initial haemoglobin levels obtained immediately after admission to the ICU. To determine the study day, we calculated the current date. The difference between the current and reference dates represents the actual study day [4].

We measured the difference in days using SAS 9.3 (SAS Corporation, Inc., Cary, NC) [5].

The following general formula was used to calculate the study day:

(1) $$\begin{array}{c} \left[\setminus \text{text}\left\{\text{Study Day}\right\}=\left(\backslash \text{text}\left\{\text{Current Date}\right\}–\backslash \text{text}\left\{\text{Reference Date}\right\}\right)+1\right]\\ \text{Study Day}=\left(31\text{ December }2023\text{ to }1\text{ October }2023\right)+1. \end{array}$$

The “+1” indicates that the reference date itself is considered Day 1.

### 2.3. Statistical Analysis

Data were collected using a chart review facilitated by electronic medical records. For statistical analysis, we used Jamovi Project, version 2.5, 2024 [3]. The patient variables are presented as the mean and standard error of mean (SE). In the second part of the analysis, the factors which could predict a drop in haemoglobin were assessed using linear mixed-effect model analysis using a reduction in haemoglobin (continuous variable) as a dependent variable and days of hospitalisation as independent variables. The data were clustered according to the number of observations. The level of significance was set at *p* < 0.05 for variable in the final model.

## 3. Results

### 3.1. Patient Characteristics

From October 2023 to December 2023, a total of 180 hospitalisations in the ICU were reviewed, and 83 (43 men, 40 women) met the inclusion criteria. Patients were characterised according to sex and age. Among men, 19 (44%) and 24 (56%) were aged < 65 and > 65 years, respectively. The mean age was 61.9 years. Among women, 17 (43%) and 23 (57%) were aged < 65 and > 65 years, respectively. The mean age was 66.3 years (Table 1).

### 3.2. Haemoglobin Concentration

**Figure 1:**
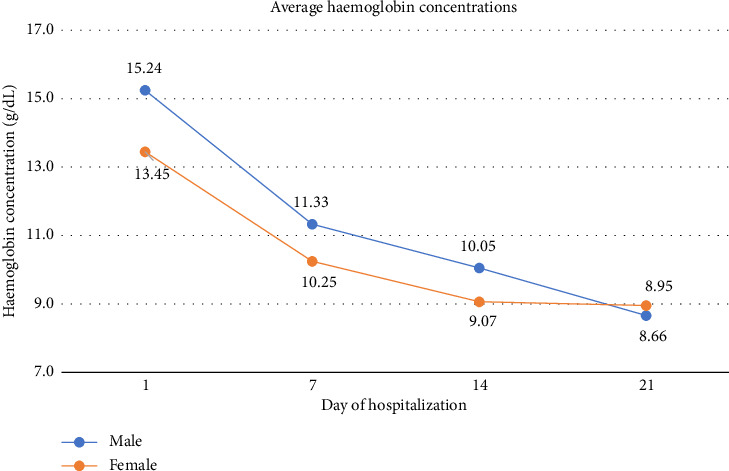
The trend of declining haemoglobin concentrations over a 21 day period of hospitalization in the critical care unit for both male and female patients [5].

Initially, the average decrease in haemoglobin during the first day of ICU hospitalisation in patients was similar in both sexes. The men had a higher average haemoglobin level (15.24 g/dL) than the women (13.45 g/dL). However, both groups experienced a significant and relatively parallel decline in haemoglobin levels from Day 7 to 14 (Figure 1). Among men, the average haemoglobin level dropped to 11.33 g/dL by Day 7 and 10.05 g/dL by Day 14. Among women, the haemoglobin level dropped to 10.25 g/dL by Day 7 and 9.07 g/dL by Day 14. On Day 21, the haemoglobin concentrations for both sexes were similar (8.95 g/dL).

The average haemoglobin concentration for the male over the 21 day period is approximately 11.39 g/dL, as for the female is approximately 10.36 g/dL, indicating that prolonged hospitalization in the critical care unit is associated with substantial reductions in haemoglobin levels for both sexes.

### 3.3. Fixed and Random Parameters Estimate

The Fixed effects estimate how much the haemoglobin level changes over time on average across all patients, regardless of individual differences. The focus is on the effect of “time in ICU” (Days 7, 14, and 21) on haemoglobin levels. We performed a linear mixed model analysis to assess the correlation between the length of hospitalisation and the decrease in haemoglobin levels (Table 2). After controlling for confounding variables such as sex, the observed differences were statistically significant. An increase in hospital stay by 1 week leads to a reduction in haemoglobin levels: Day 7 was −3.57, with an SE of 0.227. The 95% confidence interval ranges from −4.01 to −3.12. The degrees of freedom (df) are 246.0, with a t-value of −15.7 and a *p* value of less than 0.001, indicating a significant decrease in haemoglobin levels by Day 7. The estimate for Day 14 is −3.02, with an SE of 0.197. The 95% confidence interval ranges from −3.40 to −2.63. The df are 246.0, with a t-value of −15.4 and a *p* value of less than 0.001, indicating a significant decrease in haemoglobin levels by day 14. The estimate for day 21 is −2.79, with an SE of 0.185. The 95% confidence interval ranges from −3.15, −2.42. The df are 246.0, with a t-value of −15.0 and a *p* value of less than 0.001, indicating a significant decrease in haemoglobin levels by Day 21. The estimated intercept was 10.89, with a SE of 0.131. The 95% confidence interval for this estimate ranges from 10.63–11.15. The df for this estimate were 82.0, with a t-value of 83.2 and a *p* value of less than 0.001, indicating a highly significant result. The results are shown in (Table 2). These results indicated a significant decrease in haemoglobin levels as the number of hospitalisation days increased, with all estimates showing highly significant *p* values (*p* < 0.001).

The variability in haemoglobin levels among different patients was represented by an SD of 0.942 and a variance of 0.888. An ICC of 0.294 indicated that 29.4% of the total variance in haemoglobin levels could be attributed to differences between patients. The residual variability (within-patient variability) had an SD of 1.462 and variance of 2.137. There was notable variability in the declining haemoglobin levels between different patients and within individual patients over the days of ICU hospitalisation (Table 3). Approximately 29.4% of the total variability in declining haemoglobin levels was due to differences between patients, suggesting that a significant portion of the variability could be attributed to individual patient factors. A larger residual variance indicates substantial within-patient variability in haemoglobin levels over time. This reflects how patient-specific factors and time-related changes contribute to the overall variability in haemoglobin levels during ICU hospitalisation (Table 3).

## 4. Discussion

Anaemia is a condition commonly investigated in hospitalised patients. Most research has been conducted in Europe and in some countries in Asia. Several studies have shown that anaemia plays a crucial role in increasing mortality and prolonging hospital stays. To the best of our knowledge, few studies have been conducted in the Kingdom of Saudi Arabia on this topic. Therefore, this study aimed to identify the association between length of stay and the incidence of HAA in critically ill patients in the Kingdom of Saudi Arabia. A retrospective cohort study was conducted, and data from the electronic records of patients admitted to King Fahad Specialist Hospital in the Qassim region were analysed. A total of 180 ICU hospitalisations were screened. Of these, 97 (53.8%) were excluded based on the exclusion criteria, and the remaining patients (46.2%, *n* = 83) were included in the final analysis. After comparing haemoglobin values recorded on admission versus values after 1 week of admission in the ICU, all patients (100%) of both sexes and all ages had a drop in haemoglobin levels during hospitalisation. The key findings of our study were that a gradual decrease in haemoglobin was observed in all ICU patients at 1 week, 2 weeks, and 3 weeks, respectively. The largest decrease in haemoglobin levels was noted during the first week of hospitalisation. One possible explanation for this finding is that the longer the patients were hospitalised, the more likely they were to have increased amounts of blood drawn.

The results of our study are in line with other previous studies showing that length of stay is an important predictor for the development of HAA. In a recent study published by Juárez-Vela et al. in a cohort of 142 adults without iatrogenic anaemia hospitalised in the ICU, 67% had anaemia after ICU admission, slightly lower than that seen in our study. The authors commented that the prevalence of anaemia increased when patients were admitted to the ICU. Regarding the risk factors associated with the appearance of anaemia, the study findings showed that the presence of arterial and venous catheters, drains, length of stay in the ICU, and most importantly, the volume of blood removed from the critical patient were risk factors associated with the onset of anaemia [6].

A prospective observational study was conducted in a tertiary care public hospital in northern India; it included 100 patients. In this study, 67% of patients showed a decrease in haemoglobin, with the average decline being 0.37 to > 1.5 g/mL. Notably, the decrease in haemoglobin in their study was less than that observed in the current study. Similarly, a study by Ali et al. indicated that over half of the patients had some degree of anaemia and that their haemoglobin levels had deteriorated. This decline in haemoglobin levels occurred as a result of the investigated patients' exposure to multiple factors that contributed to HAA during their ICU stay [7].

In a large population-based study of critical illness survivors, anaemia was present in 41% of the participants prior to hospital admission and 80% after hospital discharge. Despite the relatively short ICU (median, 1.2 days) and hospital (median, 4.8 days) lengths of stay, the rate of incident anaemia during hospitalisation was 74%. Among critical illness survivors with accessible haemoglobin concentration data, nearly half were still anaemic 1 year after being admitted to the hospital. This group included over 25% of survivors who did not have anaemia prior to hospitalisation [8].

In contrast, in a study conducted by Villani et al., 129 patients admitted to the internal medicine department had different factors associated with the development of anaemia. They found that the haemoglobin value at admission (baseline) and the volume of blood drawn during the hospital stay were the only two factors that were statistically significant. Conversely, sex, age, chronic disease, and length of hospitalisation were not significant factors. Interestingly, the decline in Hg levels in our study was much greater than that previously observed in ICU patients and in western settings [9].

Some reports have indicated that the causes of HAA in critically ill patients include increased blood loss, impaired red cell production, and reduced red cell life span [10]. In a recent retrospective cohort study by Czempik et al. haemoglobin concentration changes over 14 days and the potential causes of iatrogenic blood loss in patients hospitalised in the ICU were analysed. The results showed that the average decrease in haemoglobin during the 7 days of ICU hospitalization in patients with anaemia was 1.2 (IQR 0.2–2.3) g/dL, while the average decrease in haemoglobin in patients without anaemia was 2.8 (IQR 1.1–3.8) g/dL. The difference between our study and this is that the majority of patients were anaemic upon ICU admission. Nevertheless, we included only patients who had a normal haemoglobin level on admission to determine the direct impact of the only admission on the decline in haemoglobin level [11].

In terms of implications for practice, our result in this study indicates that a set of patients might benefit from more frequent monitoring to avoid HAA among the patients hospitalized in the ICU in KFSH. Moreover, we propose a guide to inform health practitioners in managing blood transfers in the ICU.

Regarding implications for research, our findings from this study could support the proposed approaches to perform a large multicentre retrospective cohort study to explore the impact of medical practice on HAA in patients hospitalised in the ICU.

### 4.1. Limitations

This study has several limitations. The small sample size, retrospective design, and absence of data from patients with concurrent chronic diseases may have significantly affected our findings. Therefore, the generalisability of our findings to other critically ill populations remains unclear. Furthermore, in our study, the effect of the volume of blood drawn and patients who experienced bleeding during hospital stay on the haemoglobin drop was not investigated. In addition, we purposefully excluded patients with anaemia at the time of hospital admission to focus on the possible contribution of medical care practices to the development of anaemia. However, to the best of our knowledge our study is the first to investigate HAA in critically ill patients admitted to ICUs in the Kingdom of Saudi Arabia. As a result, more research is required to determine how hospitalisation affects the temporal trend of haemoglobin levels in patients with anaemia at the time of admission as well as the influence of concurrent haematological diseases on the development of anaemia during hospital stay.

## 5. Conclusion

In conclusion, anaemia is a common medical condition that occurs in patients admitted to the ICU. The key finding of our study was a gradual decrease in haemoglobin levels. All patients were admitted to the ICU for 1 week, 2, or 3 weeks. Most interestingly, the percentage of patients who experienced a significant decline in haemoglobin levels in our study was much higher than that in patients studied in western settings. Further large multiple ICU centre studies are needed to explore the related variables that contribute to the decline in haemoglobin levels in patients hospitalised in the ICU [12, 13].

## Figures and Tables

**Table 1:** The demographics of patients who met the inclusion and exclusion criteria [4].

Patients who met the inclusion criteria				
Sex	*N* (%)	Age		Mean
		< 65 years (%)	≥ 65 years (%)	
M	43 (51%)	19 (44%)	24 (56%)	61.9
F	40 (49%)	17 (43%)	23 (57%)	66.3

**Table 2:** The fixed effects parameter estimates of the impact of the day of hospitalization on the level of haemoglobin in patients admitted to the ICU [4].

Fixed effects parameter estimates								
Names	Effect	Estimate	SE	95% confidence interval		df	t	*p*
				Lower	Upper			
(Intercept)	(Intercept)	10.89	0.131	10.63	11.15	82.0	83.2	<0.001
Day 7	7-1	−3.57	0.227	−4.01	−3.12	246.0	−15.7	<0.001
Day 14	14-(1, 7)	−3.02	0.197	−3.40	−2.63	246.0	−15.4	<0.001
Day 21	21-(1, 7, 14)	−2.79	0.185	−3.15	−2.42	246.0	−15.0	<0.001

**Table 3:** The extent of variability in haemoglobin levels due to differences between patients and within patients over time [4].

Random components parameter estimate				
Groups	Name	SD	Variance	ICC
Patients	(Intercept)	0.942	0.888	0.294
Residual		1.462	2.137	

*Note:* Number of Obs: 332, groups: patients 83.

## Data Availability

The data that support the findings of this study are available from the electronic medical records of King Fahad Specialist Hospital in the Qassim region. Due to institutional and ethical restrictions concerning patient confidentiality, the raw data cannot be publicly shared. However, de-identified data may be made available upon reasonable request from the corresponding author and with permission from the King Fahad Specialist Hospital Ethics Committee.
